# How Different Medical School Selection Processes Call upon Different Personality Characteristics

**DOI:** 10.1371/journal.pone.0150645

**Published:** 2016-03-09

**Authors:** Nienke R. Schripsema, Anke M. van Trigt, Martha A. van der Wal, Janke Cohen-Schotanus

**Affiliations:** 1 Center for Education Development and Research in Health Professions (CEDAR), Institute for Medical Education, University of Groningen, University Medical Center Groningen, Groningen, the Netherlands; 2 Institute for Medical Education, University of Groningen, University Medical Center Groningen, Groningen, the Netherlands; Leiden University Medical Centre, NETHERLANDS

## Abstract

**Background:**

Research indicates that certain personality traits relate to performance in the medical profession. Yet, personality testing during selection seems ineffective. In this study, we examine the extent to which different medical school selection processes call upon desirable personality characteristics in applicants.

**Methods:**

1019 of all 1055 students who entered the Dutch Bachelor of Medicine at University of Groningen, the Netherlands in 2009, 2010 and 2011 were included in this study. Students were admitted based on either top pre-university grades (n = 139), acceptance in a voluntary multifaceted selection process (n = 286), or lottery weighted for pre-university GPA. Within the lottery group, we distinguished between students who had not participated (n = 284) and students who were initially rejected (n = 310) in the voluntary selection process. Two months after admission, personality was assessed with the NEO-FFI, a measure of the five factor model of personality. We performed ANCOVA modelling with gender as a covariate to examine personality differences between the four groups.

**Results:**

The multifaceted selection group scored higher on extraversion than all other groups(p<0.01), higher on conscientiousness than both lottery-admitted groups(p<0.01), and lower on neuroticism than the lottery-admitted group that had not participated in the voluntary selection process. The latter group scored lower on conscientiousness than all other groups(p<0.05) and lower on agreeableness than the multifaceted selection group and the top pre-university group(p<0.01).

**Conclusions:**

Differences between the four admission groups, though statistically significant, were relatively small. Personality scores in the group admitted through the voluntary multifaceted selection process seemed most fit for the medical profession. Personality scores in the lottery-admitted group that had not participated in this process seemed least fit for the medical profession. It seems that in order to select applicants with suitable personalities, an admission process that calls upon desirable personality characteristics is beneficial.

## Introduction

In medical school admissions, the goal is to select the applicants with the highest potential to be good doctors. In addition to possessing the appropriate medical knowledge and technical skills, the ‘ideal doctor’ has been described as someone caring, good with people, emotionally stable, and responsible.[1,2] In formal policy statements, qualities such as altruism, accountability, excellence, duty, service, integrity and respect for others are mentioned as important for professionalism in physicians.[3,4] These qualities relate to personality characteristics, which have been shown to correlate with performance in medical school and in the medical profession.[5–10] Personality is often measured with the ‘Big Five’ factor model of personality, which assesses conscientiousness, extraversion, openness to experience, agreeableness, and neuroticism.[11] Conscientiousness, which relates to dutifulness, responsibility and accountability, is the strongest and most consistent personality predictor of performance in medical school as well as in the medical profession.[8] Extraversion and openness to experience were found to be increasingly strong predictors of medical school performance over time.[12] Agreeableness, characterized by factors such as altruism and being good with people, seems beneficial for communication and collaboration skills in doctors.[9] Neuroticism, which relates to emotional instability, was found to correlate with stress, exhaustion, and dissatisfaction with medicine as a career.[13] Based on these relationships, it may be beneficial for medical schools to look for ways to select applicants who score low on neuroticism, and high on the other factors in the Big Five factor model of personality.

In order to admit applicants with such a personality profile, it would make sense to incorporate personality tests in the selection process. However, research shows that it is possible to be too conscientious, or too emotionally stable. One study indicated that relationships between these traits and job performance are not, as generally assumed in the literature, linear, but curvilinear, with the relationship disappearing at higher personality scores.[14] Selecting the applicants with the lowest scores on neuroticism and highest scores on the other Big Five factors might therefore not result in the admission of students with the most suitable personality traits. A profile of an ‘ideal’ doctor or ‘ideal’ personality values for medical workers is not available. Therefore, the use of personality tests for selection purposes carries the risk to be ineffective. Moreover, during the selection process, applicants tend to give socially desirable responses in order to increase their chances of admission.[1,15,16] In one study, the authors administered a personality test during the selection process and repeated this a few months after admission. In the test during selection, students scored significantly lower on neuroticism and higher on the other Big Five factors than after having been accepted.[1] This ‘faking good’ behaviour makes the results of personality tests administered during selection unreliable and may lead to a distortion of correlations between personality measurements and later performance.

Still, in light of the relations between personality and later performance, it would be desirable to design a selection process which rewards constructive personality characteristics and in some way, sanctions disruptive personality traits. Roughly, medical school admissions processes can be divided in processes focusing on either academic criteria such as previous academic performance, or ‘non-academic’ criteria such as professionalism, empathy and communication skills, or random selection through a lottery. Studies examining the link between these different types of selection processes and performance show that students admitted based on a top pre-university GPA perform well in the academic as well as the ‘non-academic’ domain of medical training.[17] Students accepted in a time-consuming voluntary selection process focused on several academic and ‘non-academic’ skills were shown to have a lower risk of dropout than lottery-admitted students and to outperform them in clerkships.[18,19] Additionally, participation–i.e. investment of time and effort–in a voluntary selection process seems to predict higher academic performance than lottery, regardless of the outcome of the voluntary process (acceptance or rejection). We wondered whether these performance differences could be explained by more constructive personality characteristics in the groups admitted based on top pre-university performance or selection, than in the group that was admitted by lottery.

In this study, we therefore examined personality among students who were admitted to one medical school based on either a top pre-university GPA, a voluntary multifaceted selection process, or weighted lottery. Our research question was whether students admitted through different admissions processes have different personality profiles.

## Methods

### Context

This study was conducted at University of Groningen medical school, the Netherlands. Medical schools in the Netherlands receive approximately 8500 applications annually. University of Groningen medical school offers 410 of the total of 2780 places in Dutch medical schools. Of these 410 places, around 60 are allocated to students in the International Bachelor of Medicine, in which all courses take place in English. The remaining 350 places are allocated to students in the Dutch Bachelor. In this study, we included all 1055 students who started the Dutch Bachelor’s programme in 2009, 2010, and 2011.

### Admission processes

At the time of data collection, places at University of Groningen medical school were assigned in three steps. In the first step, applicants with a pre-university GPA of 8 or higher (on a scale where 1 = poor and 10 = excellent), were granted direct access. These account for the top 5% of pre-university graduates in the Netherlands, and around 15% of all medical school applicants. Participation in the second step was voluntary. In this step, applicants participated in a multifaceted selection process which was organized by each Dutch medical school separately. Applicants who were rejected in the second step automatically enrolled in the third step. In the third step, the remaining places were assigned through a weighted lottery, in which chances of admission increase parallel to pre-university GPA. The 3-step admission system in the Netherlands has been described in more detail in a previous study.[17]

### Multifaceted selection process

Between 2009 and 2011, the multifaceted selection process comprised two phases. Students were required to hand in a personal portfolio with information about their academic background, extracurricular activities, and a number of reflection assignments. Extracurricular activities yielded points if they had been carried out for 3,5 hours or more per week during the previous 1,5 years, for at least 5 consecutive months. In the section on reflection, applicants carried out several reflective assignments. For example, they had to reflect on statements from people in their network about their suitability for the medical profession. Evaluation of the first phase indicated that students invested between 40 and 60 hours in the development of their portfolio. After scoring the portfolios, the 225 highest scoring applicants were invited for the second phase.

The second phase consisted of a full day programme with assignments in four 90-minute blocks: a writing assignment on policies in the medical field, a patient-lecture with subsequent assignments, an assignment on scientific reasoning, and an MMI-like[20] series of short interviews and role-plays. In the writing assignment, ethical decision-making and writing skills were assessed. Applicants were asked to write an essay on real-life societal dilemmas, for example China’s one-child policy. Assessment criteria were clarity, structure, quality of arguments and consistency. In the patient lecture, medical knowledge, analytical skills and professional integrity were assessed through questions about the lecture and videos depicting a physician in a professional situation. Applicants were asked about professional reactions to difficult situations in the medical field. Assessment criteria were quality and appropriateness of the arguments, insight on the micro, meta, and macro level, clarity, and consistency. In the scientific reasoning assignment, applicants answered questions about a scientific article that they were provided with. Assessment criteria were scientific reasoning skills and basic knowledge of the natural sciences. Variables that were assessed in the MMI-like block of short interviews were communication skills, collaboration skills and reflection.[17] These skills were assessed on a 7-point Likert scale by multiple independent raters. For all assignments, time-pressure and levels of difficulty were high. Applicants were ranked based on their scores for the four blocks. All blocks had the same weight, except for the MMI-like block, which was weighted double in the total score. The highest-ranking applicants were offered a study place.

### Data collection

The five personality factors conscientiousness, extraversion, openness to experience, agreeableness, and neuroticism were assessed with an authorized version of the Neo Five Factor Inventory (NEO-FFI) published in 1992 by Costa & McCrea.[21] This questionnaire consists of 60 items rated on a 5-point Likert scale ranging from 1 (strongly disagree) to 5 (strongly agree), and can be completed in approximately 10 minutes.

First-year students were asked to fill out various questionnaires on learning styles, personality, positive outcomes, and study strategies approximately two months after admission, as part of a longitudinal professionalism course. Personality was assessed with the NEO-FFI, a validated shortened version of the NEO-Pi-R, which measures the Big Five personality traits. This shortened version was used as to not burden the students too much with the questionnaires during their studies. The outcomes of the inventory were communicated to the students. Students wrote a reflection report on the relevance of the outcomes for their study behaviour, how they planned to use beneficial aspects of their personality to their advantage, and how they would manage aspects that might lead to problems. In order to answer the research question for this study, we focused on the personality data. The data were derived from the university administration and then anonymized.

### Participants

Of all 1055 students who were admitted to the Dutch Bachelor of Medicine at University of Groningen in 2009, 2010 and 2011, 1019 students (response rate = 96,6%; mean age at the start of the first year = 18.6; 69% females) filled out the NEO-FFI. We analysed personality in four groups: students who were admitted based on a pre-university GPA ≥ 8 (n = 139; mean age at the start of the first year = 18.0; 70% females); students who were accepted in the multifaceted selection process (n = 286; mean age at the start of the first year = 18.5; 74% females); lottery-admitted students who had been initially rejected in the multifaceted selection process (n = 310; mean age at the start of the first year = 18.5; 69% females); and lottery-admitted students who had not participated in this process (n = 284; mean age at the start of the first year = 19.1; 63% females) (Table 1).

**Table 1:** Descriptive statistics for percentages of females and mean age at the start of the first year for the four student groups.

	N	% females^i^	Mean age^ii^
Top pre-university GPA	139	71	18.0
Multifaceted selection process	286	74	18.5
Selection-rejected lottery	310	69	18.5
Lottery	284	63	19.1
Total	1019	69	18.6

^i^ Multifaceted selection process > Lottery (p<0.05)

^ii^ Top pre-university GPA < other groups (p<0.01); Lottery > other groups (p<0.001)

### Data analysis

Mean personality factor score differences between male and female students were analysed with independent samples t-tests. Mean differences between the four groups were analysed using one-way analysis of covariance (ANCOVA). Gender was added as a covariate in all ANCOVA analyses because the t-tests showed that gender was related to personality factor scores. After initial analyses, the interaction term of gender and group was deleted from all models because this interaction was non-significant for all personality factors. This resulted in a model in which main effects of gender and group on personality factor scores were analysed. When ANCOVA analyses showed significant group differences, Bonferroni post hoc multiple comparison analyses were performed to assess which specific groups differed.

### Data handling and permission

University of Groningen privacy policy states that student records may be used for research purposes, given that reports cannot be traced back to individual students.[22] Data were derived from the university administration and anonymized in accordance with this privacy policy.

This study received ethics approval of the Ethical Review Board of the Dutch Association for Medical Education (NERB dossier number: 359).

## Results

### Descriptive statistics

The percentage of female students was higher in the group that was accepted in the multifaceted selection procedure than in the lottery group that had not participated in this process (p<0.05). In comparison with the other groups, mean age was lower in the top pre-university group (p<0.01) and higher in the lottery group that had not participated in the multifaceted selection process (p<0.001) (Table 1).

Independent samples t-tests showed that mean personality factor scores among female students were significantly higher than among male students for neuroticism (Mean difference MD = 2.98; SE = 0.46; t_1017_ = 6.46; p<0.001), agreeableness (MD = 3.75; SE = 0.30; t_1017_ = 12.32; p<0.001), and conscientiousness (MD = 2.98; SE = 0.40; t_1017_ = 7.49; p<0.001). In further analyses, gender was therefore added as a covariate.

Mean personality factor scores for the four student groups are displayed in Table 2. The ANCOVA models comparing the groups’ personality factor scores are displayed in Table 3.

**Table 2:** Mean Big Five personality factor scores for total and the four student groups.

	Top pu* (SE)	MFSP* (SE)	SR-Lottery* (SE)	Lottery (SE)
Conscientiousness	45.8 (0.52)	46.0 (0.33)	44.3 (0.33)	43.0 (0.37)
Extraversion	43.2 (0.46)	45.8 (0.28)	44.4 (0.29)	44.0 (0.32)
Openness to experience	39.8 (0.49)	39.2 (0.31)	38.4 (0.32)	39.0 (0.35)
Agreeableness	46.1 (0.44)	45.9 (0.28)	45.3 (0.25)	44.5 (0.29)
Neuroticism	29.7 (0.55)	29.7 (0.39)	30.1 (0.41)	31.4 (0.43)
N	139	286	310	284

* Top pu = top pre-university GPA group; MFSP = Multifaceted accepted group; SR-Lottery = Selection-rejected lottery group

**Table 3:** ANCOVA analyses of mean differences in Big Five personality factor scores between the four student groups with gender as a covariate.

	F_group_	p_group_	Post hoc for groups*	MD**	SE_MD_	p_MD_
Conscientiousness	F_3, 1014_ = 12.78	0.000	MFSP > SR-Lottery	1.73	0.47	0.002
			MFSP > Lottery	3.02	0.48	0.000
			Lottery < Top pu	-2.81	0.60	0.000
			Lottery < SR-Lottery	-1.30	0.47	0.038
Extraversion	F_3, 1014_ = 10.24	0.000	MFSP > Top pu	2.60	0.53	0.000
			MFSP > SR-Lottery	1.38	0.42	0.006
			MFSP > Lottery	1.84	0.43	0.000
Openness to experience	F_3, 1014_ = 2.17	0.090	NS			
Agreeableness	F_3, 1014_ = 3.70	0.011	Top pu > Lottery	1.60	0.46	0.003
			MFSP > Lottery	1.38	0.37	0.001
Neuroticism	F_3, 1014_ = 4.67	0.003	MFSP < Lottery	-1.62	0.57	0.025

* Top pu = top pre-university GPA group; MFSP = Multifaceted accepted group; SR-Lottery = Selection-rejected lottery group

** MD = Mean difference

### Conscientiousness

Mean conscientiousness scores differed between groups (F_3, 1014_ = 12.78; p<0.001). Bonferroni post hoc multiple comparison analyses showed that the group that was accepted in the multifaceted selection process had a higher mean conscientiousness score than both lottery groups (MD: 1.73–3.02; SE:0.47–0.48; p<0.01). The lottery group that had not participated in the multifaceted selection process had a lower mean conscientiousness score than the top pre-university group and the selection-rejected lottery group (MD: -2.81– -1.30; SE:0.47–0.60; p<0.05).

### Extraversion

Mean extraversion scores differed between groups (F_3, 1014_ = 10.24; p<0.001). The group that was accepted in the multifaceted selection process had a higher mean extraversion score than all other groups (MD: 1.38–2.60; SE:0.42–0.53; p<0.01).

### Openness to experience

The four groups did not differ significantly in mean openness to experience scores (F_3, 1014_ = 2.17; p>0.05).

### Agreeableness

Groups differed in mean agreeableness scores (F_3, 1014_ = 3.70; p<0.05). The top pre-university group and the group that was accepted in the multifaceted selection process had higher mean agreeableness scores than the lottery group that had not participated in the multifaceted process (MD: 1.38–1.60; SE:0.37–0.46; p<0.01).

### Neuroticism

Groups differed in mean neuroticism scores (F_3, 1014_ = 4.67; p<0.001). The mean neuroticism score in the group that was accepted in the multifaceted selection process was lower than in the lottery-admitted group that had not participated in this process (MD = -1.62; SE = 0.57; p<0.05).

## Discussion

We found small, but significant differences in personality factor scores between the different admission groups. Personalities in the group that was accepted in the voluntary multifaceted selection process seemed most fit for the medical profession, with higher conscientiousness scores than both lottery-admitted groups, higher extraversion scores than all other groups, and higher agreeableness and emotional stability scores than the lottery-group that had not participated in the selection process. Personality scores in the lottery-admitted group that had not participated in the multifaceted selection process seemed least fit.

The group accepted in the multifaceted selection process scored lower on neuroticism than students who were admitted through lottery without having participated in the selection process. This is in line with earlier research showing significantly lower neuroticism scores among students who were admitted based on an interview than among students who were admitted based on a non-interview process.[23] It is possible that the voluntary nature of the selection processes induces self-selection of emotionally stable applicants. The stress of an entire day of assessments might affect applicants who are less emotionally stable more strongly than other applicants, and cause them to wait for the lottery. The high conscientiousness scores among selection-accepted students compared to both lottery-admitted groups may be explained by the requirements for the multifaceted selection process and its voluntary nature. Applicants are required to invest approximately 40 to 60 hours in completing their first-phase portfolio. This may discourage less conscientious applicants, who might spend less time on their portfolio or put less effort into the assignments. This may cause them to score lower in the selection process, as in addition to the evaluation of quality, the assignments are evaluated in terms of completeness, orderliness and rigour. In the lottery-admitted group that had not participated in the selection process, the large investment of time and effort that is required might have caused applicants to shy away from participation. The MMI-like[20,24,25] series of interviews and role-plays in the second phase of the selection process, in which interpersonal skills are of high importance, might explain the high scores on extraversion in the selection-accepted group. MMI-scores were indeed found to be positively related to this factor.[26,27]

The group admitted through lottery without having participated in the voluntary selection process had the least suitable personality scores. Conscientiousness scores in this group were lower than among lottery-admitted students who had participated in the voluntary multifaceted selection process but were initially rejected. Again, the time investment that is required for participation might offer an explanation. Another explanation might be that participation in the multifaceted selection process may be related to motivation,[17,28] which in turn relates to personality. A meta-analysis of the relationship between personality and performance motivation showed that conscientiousness was positively related to performance motivation (goal-setting, expectancy, and self-efficacy motivation).[29] This effect might be apparent in the group that participated in the multifaceted selection process as well. However, this same meta-analysis showed that neuroticism was inversely related to performance motivation,[29,30] whereas in our study, we did not find a significant difference between the two lottery-admitted groups on neuroticism scores. As motivation is not only related to personality but also to performance in medical school,[31] it would be worthwhile to investigate the role of motivation in the relation between personality and performance in future research.

Psychology research indicates that relations between job performance and personality characteristics are curvilinear. Too much conscientiousness or emotional stability does not seem to contribute to better performance.[14] One could therefore argue that our conclusions about the ‘fit’ between personalities in the different groups and the medical profession are unsupported, because there is no indication of ‘ideal’ values of the personality traits. However, rather than measuring personality with a personality test during selection, we assessed whether the different admissions processes called upon different personality characteristics *indirectly*. Extremely high or low scores on the personality traits would likely not result in suitable behaviour, and would therefore probably lead to lower scores in pre-university education or in the selection process. For example, through rewarding suitable behaviour, the multifaceted selection process may select out those applicants who are too conscientious, or too emotionally stable. Additionally, previous findings showed that performance was better among students who were admitted based on a top pre-university GPA or multifaceted selection than among lottery-admitted students.[17] It thus seems that in these groups, students have constructive personality traits. In choosing a selection process, medical schools should therefore be aware of this indirect selection on personality traits. Taking this a step further, medical schools may modify the focus of their selection process to call upon desirable personality traits, such as conscientiousness.

We found significant personality differences between the four groups, but the differences were small. This might be explained by the fact that the pool of medical school applicants is quite homogeneous, as all applicants are part of a highly educated group with similar vocational interests.[32] In general, medical students seem to have different personality profiles than other students. Medical students score high on extraversion and agreeableness compared to students in sciences or applied sciences.[9] In such a homogeneous group, effect sizes of different admissions processes are bound to be small. The fact that we did find group differences in personality scores indicates that the choice for a certain selection process might affect the student population in terms of personality.

Our study offers a unique insight into personality characteristics relevant in different medical school selection processes. However, some limitations should be taken into account when interpreting our results. First, we used the NEO-FFI inventory, a shortened version of the original NEO-PI-R five factor model. This shortened version measures only the five main personality factors, whereas the original test also assesses different facets within these higher-level factors. In further research, it could be beneficial to use the original questionnaire, as despite being strongly related to each other, the facets within the main personality factors seem to have different, sometimes even inversed, correlations with workplace outcomes.[26] For example, it was found that half the facets of openness to experience relate positively to job performance, whereas the other half correlate negatively.[33] This might explain our non-significant results for this factor as well. Second, there is an ongoing debate on the stability of personality characteristics in this age group. Some argue that personality characteristics are stable in adulthood, whereas others argue that personality characteristics are susceptible to change, depending on environmental, physical, and cognitive factors.[34] If personality characteristics are indeed still changing in adulthood, this affects the reliability of our measure of personality: conclusions could be different when personality in the same groups is measured again after a period of time. A third limitation of our study is that conducting the research at a single medical school might lead to a selection bias, as applicants generally apply to a specific medical school based on their personal preferences. For example, a study by Wilson and colleagues (2013) showed that higher agreeableness and conscientiousness scores were associated with attending an undergraduate school and a rural or community focused school.[35] It therefore remains unclear whether our results are generalizable to other medical schools within the same admission system.

### Conclusions

Students accepted in the voluntary multifaceted selection process seemed to have the most suitable personality profiles. Lottery-admitted students who had not first participated in this process had the least suitable personality profiles. In the design of an admissions process, medical schools should take into account that different selection tools might call upon different personality traits. They may use this effect to select applicants with suitable personalities.

## References

[1] Griffin B, Wilson IG. Faking good: self-enhancement in medical school applicants. Med Educ. 2012;46(5):485–490. 10.1111/j.1365-2923.2011.04208.x 22515756

[2] Powis D. Personality testing in the context of selecting health professionals. Med Teach. 2009;31(12):1045–1046. 10.3109/01421590903390601 19995165

[3] American Board of Internal Medicine. Project Professionalism. 1995.

[4] General Medical Council. Good medical practice. 2013.

[5] Doherty EM, Nugent E. Personality factors and medical training: a review of the literature. Med Educ. 2011;45(2):132–140. 10.1111/j.1365-2923.2010.03760.x 21208259

[6] Ferguson E, Sanders A. Predictive validity of personal statements and the role of the five-factor model of personality in relation to medical training. J Occup Organ Psychol. 2000;73:321–344.

[7] Haight SJ, Chibnall JT, Schindler DL, Slavin SJ, MEd. Associations of Medical Student Personality and Health/Wellness Characteristics With Their Medical School Performance Across the Curriculum. Acad Med. 2012;87(4):476–485. 10.1097/ACM.0b013e318248e9d0 22361792

[8] Hojat M, Erdmann JB, Gonnella JS. Personality assessments and outcomes in medical education and the practice of medicine: AMEE Guide No. 79. Med Teach. 2013;35(7):1267–e1301. 10.3109/0142159X.2013.785654 23614402

[9] Lievens F, Coetsier P, De Fruyt F, De Maeseneer J. Medical students' personality characteristics and academic performance: a five-factor model perspective RID A-3083-2009. Med Educ. 2002;36(11):1050–1056. 12406265 10.1046/j.1365-2923.2002.01328.x

[10] Lievens F, Ones DS, Dilchert S. Personality Scale Validities Increase Throughout Medical School. J Appl Psychol. 2009;94(6):1514–1535. 10.1037/a0016137 19916659

[11] McCrae RR, John OP. An Introduction to the Five-Factor Model and Its Applications. J Pers. 1992;60(2):175–215. 10.1111/j.1467-6494.1992.tb00970.x 1635039

[12] Lievens F, Ones DS, Dilchert S. Personality Scale Validities Increase Throughout Medical School. J Appl Psychol. 2009;94(6):1514–1535. 10.1037/a0016137 19916659

[13] McManus IC, Keeling A, Paice E. Stress, burnout and doctors' attitudes to work are determined by personality and learning style: a twelve year longitudinal study of UK medical graduates. BMC Med. 2004;2:29 15317650 10.1186/1741-7015-2-29 PMC516448

[14] Le H, Oh I, Robbins SB, Ilies R, Holland E, Westrick P. Too much of a good thing: Curvilinear relationships between personality traits and job performance. J Appl Psychol. 2011;96(1):113–133. 10.1037/a0021016 20939656

[15] Albanese M, Snow M, Skochelak S, Huggett K, Farrell P. Assessing personal qualities in medical school admissions. Acad Med. 2003;78(3):313–321. 12634215 10.1097/00001888-200303000-00016

[16] Griffin B, Hesketh B, Grayson D. Applicants faking good: evidence of item bias in the NEO PI-R. Pers Individ Dif. 2004;36(7):1545–1558.

[17] Schripsema NR, van Trigt AM, Borleffs JCC, Cohen-Schotanus J. Selection and study performance: comparing three admission processes within one medical school. Med Educ. 2014;48(12):1201–1210. 10.1111/medu.12537 25413913

[18] Urlings-Strop LC, Stijnen T, Themmen APN, Splinter TAW. Selection of medical students: a controlled experiment. Med Educ. 2009;43(2):175–183. 10.1111/j.1365-2923.2008.03267.x 19161489

[19] Urlings-Strop LC, Themmen APN, Stijnen T, Splinter TAW. Selected medical students achieve better than lottery-admitted students during clerkships. Med Educ. 2011;45(10):1032–1040. 10.1111/j.1365-2923.2011.04031.x 21883405

[20] Eva K, Rosenfeld J, Reiter H, Norman G. An admissions OSCE: the multiple mini-interview. Med Educ. 2004;38(3):314–326. 14996341 10.1046/j.1365-2923.2004.01776.x

[21] Costa PT, McCrae RR. Revised NEO Personality Inventory (NEO-PI-R) and the Five Factor Inventory (NEO-FFI): Professional Manual. Odessa, Florida: Psychological Assessment Resources; 1992.

[22] University of Groningen. Regulations concerning the Protection of Personal Data. July 3, 2015; Available at: http://www.rug.nl/about-us/organization/rules-and-regulations/algemeen/regeling-bescherming-persoonsgegevens, January 22, 2016.

[23] Azman MA, Yaacob NA, Yusoff MSB, Noor SH. Comparative Study on Medical Student Personality Traits between Interview and Non-interview Admission Method in University Sains Malaysia. Procedia Soc Behav Sci. 2014;116:2281–2285.

[24] Eva KW, Macala C. Multiple mini-interview test characteristics:? tis better to ask candidates to recall than to imagine. Med Educ. 2014;48(6):604–613. 10.1111/medu.12402 24807436

[25] Eva KW, Reiter HI, Trinh K, Wasi P, Rosenfeld J, Norman GR. Predictive validity of the multiple mini-interview for selecting medical trainees. Med Educ. 2009;43(8):767–775. 10.1111/j.1365-2923.2009.03407.x 19659490

[26] Griffin B, Wilson I. Associations between the big five personality factors and multiple mini-interviews. Adv Health Sci Educ Theory Pract. 2012;17(3):377–388. 10.1007/s10459-011-9316-1 21751103

[27] Jerant A, Griffin E, Rainwater J, Henderson M, Sousa F, Bertakis KD, et al Does Applicant Personality Influence Multiple Mini-Interview Performance and Medical School Acceptance Offers? Acad Med 2012;87(9):1250–1259. 22836836 10.1097/ACM.0b013e31826102ad

[28] Wouters A, Bakker A, van Wijk I, Croiset G, Kusurkar R. A qualitative analysis of statements on motivation of applicants for medical school. BMC Med Educ. 2014;14(1):200. 25248374 10.1186/1472-6920-14-200 PMC4180534

[29] Judge TA, Ilies R. Relationship of personality to performance motivation: A meta-analytic review. J Appl Psychol. 2002;87(4):797–807. 12184582 10.1037/0021-9010.87.4.797

[30] Wright TA. What Every Manager Should Know: Does Personality Help Drive Employee Motivation? Acad Manage Exec. 2003;17(2):131–133.

[31] Rhoads JM, Gallemore JLJ, Gianturco DT, Osterhout S. Motivation, medical school admissions, and student performance. J Med Educ. 1974;49(12):1119–1127. 4427334 10.1097/00001888-197412000-00002

[32] Siu E, Reiter HI. Overview: what's worked and what hasn't as a guide towards predictive admissions tool development. Adv Health Sci Educ Theory Pract. 2009;14:759–75. 10.1007/s10459-009-9160-8 19340597

[33] Griffin B, Hesketh B. Why openness to experience is not a good predictor of job performance. Int J Select Assess. 2004;12(3):243–251.

[34] Caspi A, Roberts B, Shiner R. Personality development: Stability and change. Annu Rev Psychol. 2005;56:453–484. 15709943 10.1146/annurev.psych.55.090902.141913

[35] Wilson I, Griffin B, Lampe L, Eley D, Corrigan G, Kelly B, et al Variation in personality traits of medical students between schools of medicine. Med Teach. 2013;35(11):944–948. 10.3109/0142159X.2013.827331 24001304

